# Holistic Valorization of Hemp through Reductive Catalytic Fractionation

**DOI:** 10.1021/acssuschemeng.1c06607

**Published:** 2021-12-15

**Authors:** Suthawan Muangmeesri, Ning Li, Dimitrios Georgouvelas, Pierre Ouagne, Vincent Placet, Aji P. Mathew, Joseph S. M. Samec

**Affiliations:** † Department of Organic Chemistry, Stockholm University, 106 91 Stockholm, Sweden; ‡ State Key Laboratory of Catalysis (SKLC), Dalian National Laboratory for Clean Energy (DNL), Dalian Institute of Chemical Physics (DICP), Dalian 116023, People’s Republic of China; § Department of Materials and Environmental Chemistry, Stockholm University, 106 91 Stockholm, Sweden; ∥ Laboratoire Génie de Production, Université de Toulouse, ENIT, 65016 Tarbes, France; ⊥ Department of Applied Mechanics, Univ. Bourgogne Franche-Comté, FEMTO-ST Institute, UFC/CNRS/ENSMM/UTBM, F-25000 Besançon, France

**Keywords:** Hemp hurd, Lignin, Biomass valorization, Reductive catalytic fractionation, Organosolv pulping, Dissolving pulp, Nanocellulose, Monophenolic compounds

## Abstract

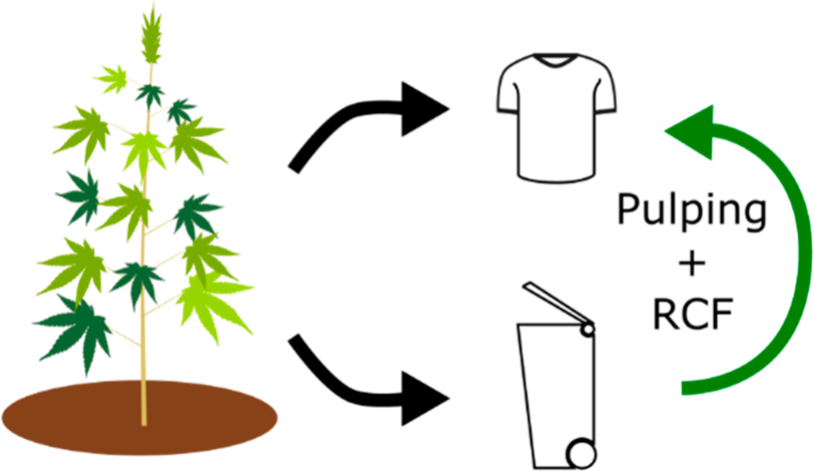

Despite the increased use of hemp fiber, negligible attention has been given to upgrade the hemp hurd, which constitutes up to 70 wt % of the hemp stalk and is currently considered a low-value byproduct. In this work, valorization of hemp hurd was performed by reductive catalytic fractionation (RCF) in the presence of a metal catalyst. We found an unexpectedly high yield of monophenolic compounds (38.3 wt %) corresponding to above 95% of the theoretical maximum yield. The high yield is explained by both a thin cell wall and high S-lignin content. In addition, organosolv pulping was performed to generate a pulp that was bleached to produce dissolving-grade pulp suitable for textile fiber production (viscosity, 898 mL/g; ISO-brightness, 90.2%) and nanocellulose. Thus, we have demonstrated a novel value chain from a low-value side stream of hemp fiber manufacture that has the potential to increase textile fiber production with 100% yield and also give bio-oil for green chemicals.

## Abstract 2

Hemp hurd, a byproduct from hemp fiber production, was successfully valorized by reductive catalytic fractionation to give high yields of monophenolic compounds and a pulp suitable for textiles or nanocellulose.

## Introduction

Natural fibers are massively cultivated from plants, especially cotton, which is widely grown as a global economic crop mainly for textile manufacture.^1^ Cotton fields cover 34.5 million (M) hectares (ha) of arable land worldwide with an average yield of 2.14 tons ha^–1^ yr^–1^ seed cotton, corresponding to a global average annual production of cotton lint of 0.76 tons ha^–1^ yr^–1^.^2^ Even though cotton is a bio-based fiber, its sustainability is questionable.^3^ This is due to the high maintenance cost of growing cotton, which demands large amounts of pesticides and chemical fertilizers derived from fossil oil.^4,5^ In addition, around 3 tons of water is consumed for each ton of cotton produced.^6^ Measures have been made to tackle these problems such as genetic modification, site selection, and nonchemical control strategies. However, cotton production is still debated.^7−9^ This has led to an effort to replace cotton with bast fiber crops such as flax and hemp that do not require the same maintenance.^10,11^

Hemp (*Cannabis Sativa*) is a herbaceous crop that can be grown in most climates, while cotton is limited to cultivation in subtropical climate zones. Hemp has recently gained attention for natural fiber production due to both agricultural and sustainability reasons. This includes resistance to drought and pests, prevention of soil erosion, and less water demand in comparison to other fiber crops. It can supply both phytochemical and lignocellulosic biomass applications.^12^ From a phytochemical perspective, hemp produces various chemicals such as phenolic compounds, terpenes, and cannabinoids which can be utilized in the pharmaceutical industry.^13^ The hemp stem composes of approximately 30 wt % of outer bast fiber and 70 wt % of the inner core (woody part also known as hurd or shiv).^14^ The two fractions must be separated to be further utilized. Hemp fiber extraction starts with retting, where pectin is digested by natural microorganisms and the inner core is separated from the fiber. A mechanical process is then applied to break down the stem using fluted rolls followed by scutching, which separates the fiber from the hurd core. The final step is hackling, also called decortication, to comb the fiber and remove unwanted particles.^15^ The hemp fiber is predominately utilized for the textile industry, insulation material, and production of bioplastics in the automotive industry.^16,17^ However, the hemp hurd, which constitutes 70 wt % of the hemp stalks, is considered a byproduct from textile fiber production and is only used for low-value applications, such as animal bedding due to its high absorption capacity, and as a concrete additive. Due to the supply exceeding the demand, the excessive waste of hemp hurd is currently disposed by combustion and landfill accumulation.^18^ There are few examples of research that utilize hemp hurd. In most cases, the main focus has been on material applications such as antibacterial, biocomposite, and activated carbon materials.^19−21^

Reductive catalytic fractionation (RCF)^22−27^ is a promising strategy that integrates lignin depolymerization with biomass fractionation using heterogeneous catalysis. It produces lignin-derived aromatic monomers and oligomers along with carbohydrate pulps and sugars. In general, RCF can be performed in batch and flow-through systems in the presence of a metal catalyst, hydrogen donor, and a mixture of aqueous and organic solvents.

**Scheme 1:**
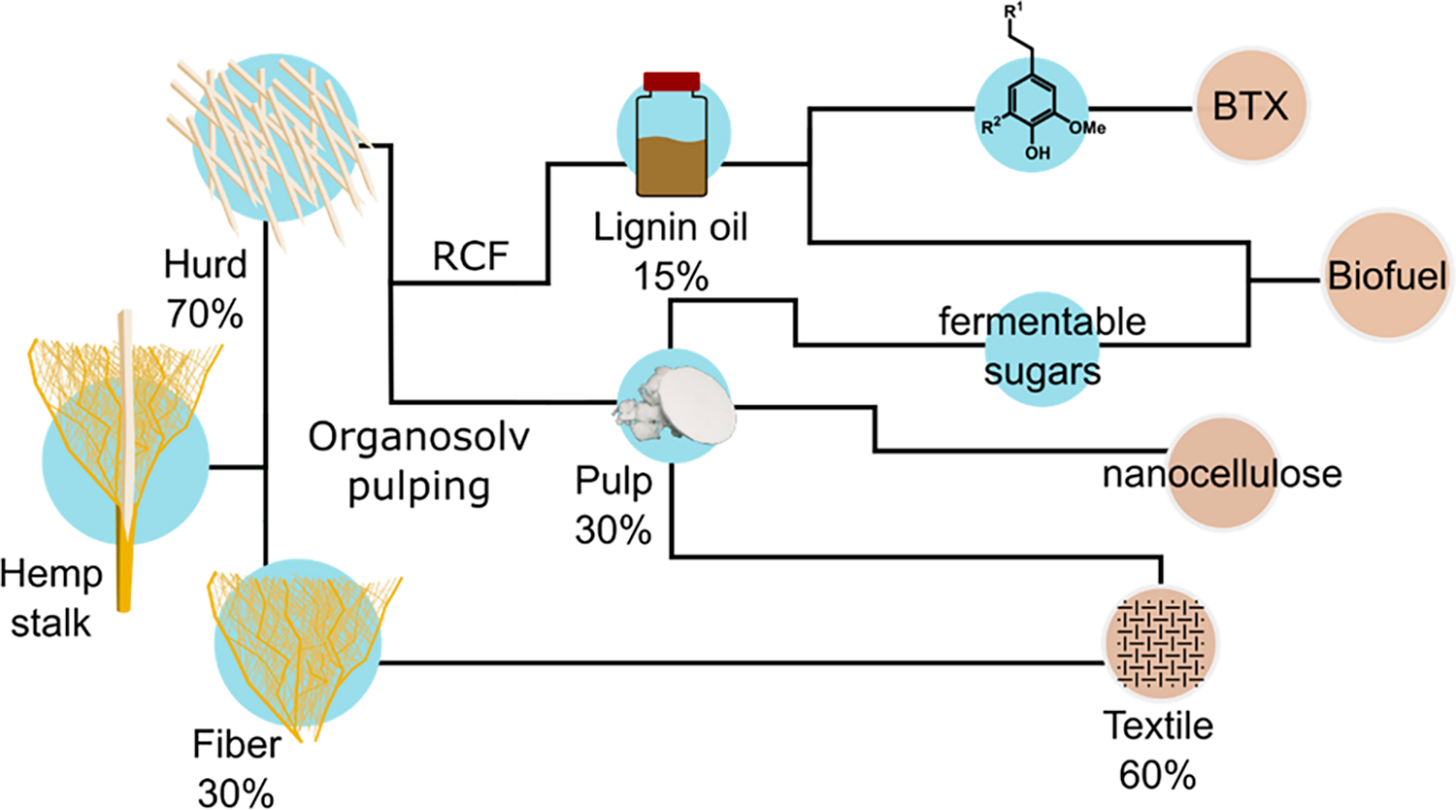
Hemp Hurd Valorized by RCF to Yield a Lignin Oil and Organosolv Pulping to Yield Textile Fiber

In this work, valorization of hemp hurd by organosolv pulping has been performed in a batch system to yield a high-value pulp suitable for textile fibers and nanocellulose.^28,29^ By the application of RCF, a lignin oil enriched in monophenolic compounds was generated that could be used for bulk chemical production such as BTX (Scheme 1).^30−32^ In recent findings using flow-through systems, pulp can be obtained without the contamination of the catalyst together with the lignin oil.^33−35^ However, for this preliminary study of this herbaceous type biomass, a flow-through methodology was not our focus. However, this will be of interest in future studies. Instead, we performed organosolv pulping and were able to increase the overall yield of fiber for textiles production by 100%.

## Results and Discussion

### RCF of Hemp Hurd

RCF was performed in a steel pressure reactor using a transition-metal catalyst (Pd/C) and formic acid as the hydrogen donor. The solvent system comprised MeOH/H_2_O (7/3 v/v), and *p*-toluenesulfonic acid (*p*-TSA) 1.1 g/L was added as a hydrolysis catalyst. The reaction conditions were optimized by varying the reaction time and the amounts of formic acid. The outcome of the reactions was detected both qualitatively and quantitatively by GC-MS and GC-FID on comparison to synthesized monophenolic compounds and quantified using an internal standard. The distribution of monophenolic compounds from GC-MS after RCF is shown in Table 1. When the reaction time was increased, the yield of monophenolic compounds increased to 38.3 wt % in 4 h (entry 3). When the reaction time was increased to 8 h (entry 6), the yield of monophenolic compounds decreased, which could possibly be explained by hydrodearomatization, as it is one of the major side reactions that causes a low aromatic monomer yield.^30,36−38^ However, hydrodearomatized products were not detected by GC, probably due to the volatility of the compounds and low yields of dearomatized products (38.3% vs 34.9%). The influence of *p*-TSA was investigated. We found that the yield of monophenolic compounds decreased from 38.3 to 33.6 wt % in the absence of *p*-TSA (entry 4). This shows that solvolysis is promoted by the acid catalyst. Without formic acid, the yield of monophenolic compounds was significantly decreased to 15.9 wt % showed in Entry 2, due to the deficiency of hydrogen donors to facilitate hydrogenation/hydrogenolysis for the generation of stable monophenolic compounds. Higher concentrations of formic acid did not improve the results (Entry 5).

**Table 1:**
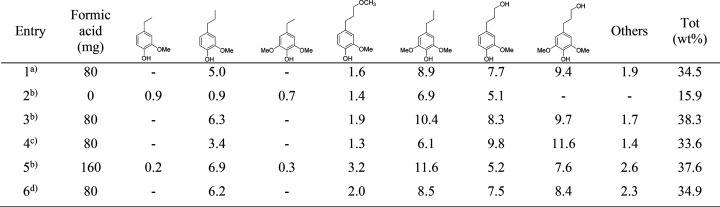
RCF Optimization and Monophenolic Yield Quantified by GC-FID

a Reaction conditions: 3 h, *p*-TSA (1.1 g/L).

b Reaction conditions: 4 h, *p*-TSA (1.1 g/L).

c Reaction conditions: 4 h, no *p*-TSA involved.

d Reaction conditions: 8 h, *p*-TSA (1.1 g/L).

**Figure 1:**
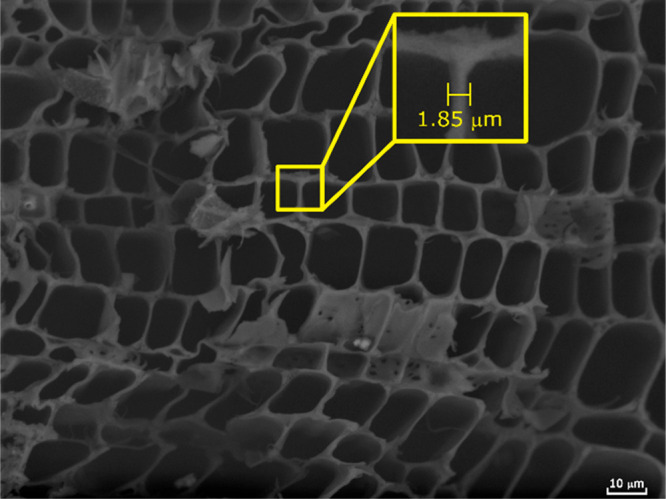
SEM image of the cross-section of hemp hurd wood chips.

RCF of hemp hurd shows an unexpectedly high monomer yield (38.3 wt %). This corresponds to above 95% of the theoretical maximum yield determined by thioacidolysis (39.7%) (see the Supporting Information). We propose that the density and particle size of biomass potentially affect lignin extraction. Recent reports have emphasized the effect of density and particle size of biomass toward lignin extraction.^39,40^ Thus, the morphology of hemp hurd was investigated by SEM (scanning electron microscopy), as shown in Figure 1. A honeycomb-like cell wall structure was observed. The average cell wall thickness was measured to be 1.95 μm (standard deviation 0.17), which is slightly thinner than that of other herbaceous crops (∼2.00–3.00 μm).^41−43^ This might explain the observed high yield of monophenolic compounds. This is rationalized by an enhancement of the intrinsic kinetics of the solvent-based fractionation, where a thinner cell wall promotes mass transfer through diffusion during solvolytic lignin extraction.^39^ Moreover, 80% delignification can be achieved after organosolv and no lignin monomer was observed (Figures S8 and S9) due to lignin recondensation.

**Figure 2:**
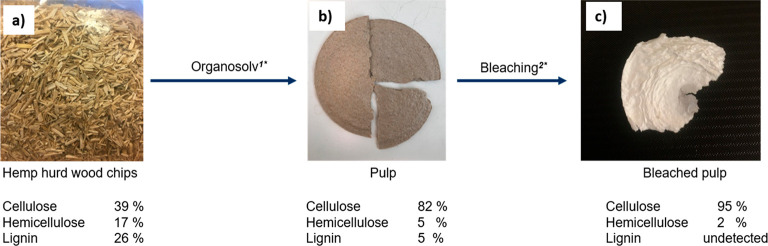
Pulping process from hemp hurd wood chips to bleached pulp with lignocellulosic composition: (a) raw material before RCF; (b) obtained pulp after organosolv pulping; (c) obtained bleached pulp after chlorite bleaching. Reaction conditions: (1*) 0.8 g of hemp hurd stick, EtOH/H_2_O (65/35) 10 mL, 100 μL of 1% HCl, 175 °C, 3 h; (2*) 6.0 g of pulp, 300 mL of 1.7% NaClO_2_ solution, 300 mL of 2.7% NaOH and 7.5% of AcOH solution, 80 °C, 2 h.

To obtain a high-quality pulp, the RCF conditions described above could not be applied due to catalyst contamination of the pulp. In this study, a separate organosolv pulping process was performed on the hemp hurd (Figure 2a) without metal catalyst to evaluate the pulp for fiber production. It should be noted that, after organosolv pulping, the cellulose fraction was isolated as a pulp (Figure 2b).^44^ Such pulps can be used in the commercial production of cellulose derivatives such as cellulose acetate, cellulose nitrate, and viscose.^28^ To meet the requirements for these applications, a pulp with both a high viscosity and brightness is required. Thus, chlorite bleaching was applied to remove the remaining lignin residues to give the white pulp shown in Figure 2c with a high viscosity (898 mL/g) and high ISO-brightness (90.2%), which meets the requirements for viscose production.

### Nanocellulose

**Figure 3:**
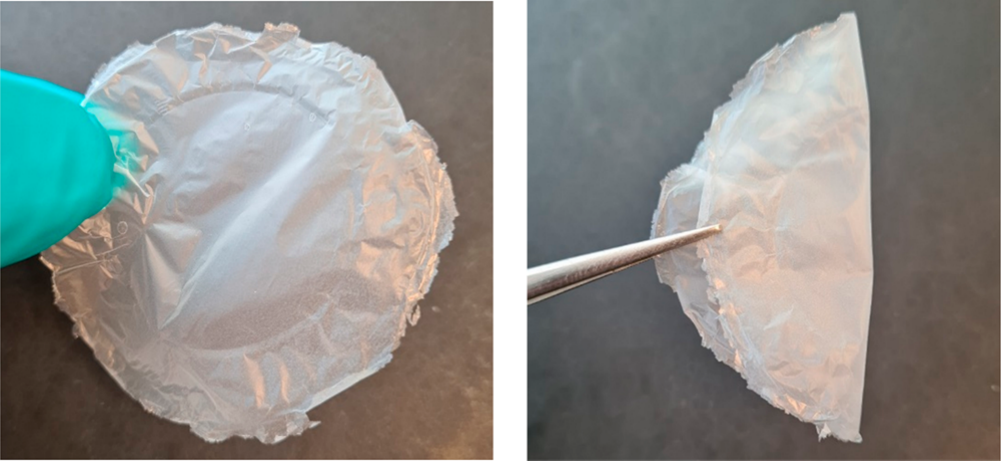
Colloidal dispersion of hemp hurd CNF.

**Figure 4:**
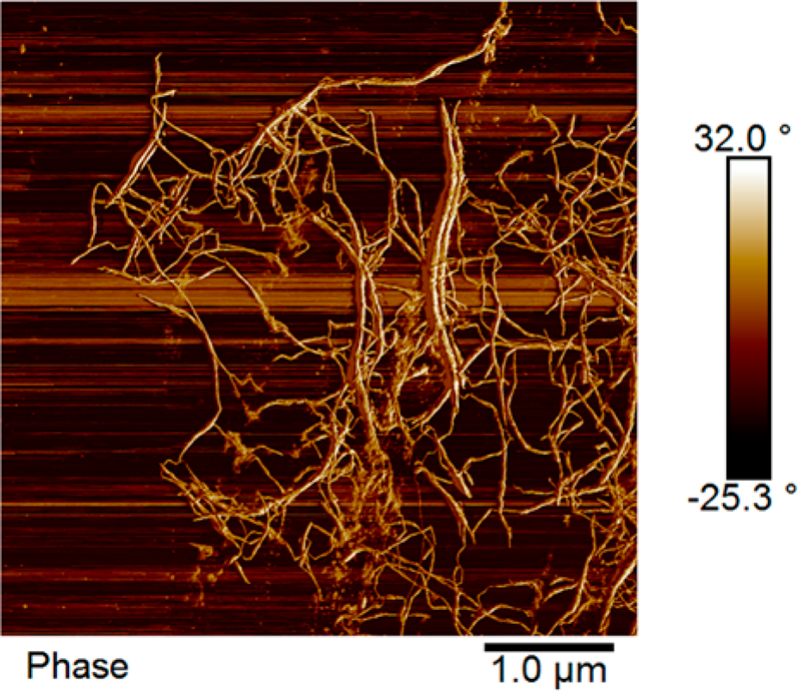
AFM image of hemp hurd CNF.

Nanocellulose is a general class of materials that can be subdivided into three groups: cellulose nanocrystals (CNC), cellulose nanofibers (CNF), and bacterial cellulose (BC).^45^ The main difference, which also leads to morphological variations among these three groups, is the production method of each one of them. Generally, CNC is derived from chemical treatment of cellulose sources (acid hydrolysis or oxidation), CNF is obtained from the delamination of cellulose pulps with mechanical treatment (using high-pressure homogenizers, microfluidizers, or grinders), and BC is produced by bacteria.^46,47^ Due to its high aspect ratio and fiber entanglement, CNF has been used as fillers and reinforcement in nanocomposites and/or hybrids in order to not only improve the mechanical properties (i.e., tensile strength, and Young’s modulus) but also enhance the thermal stability of other biopolymers (i.e., chitosan) or lower the swelling degree and water solubility of synthetic polymers: for instance, poly(vinyl alcohol) films.^47^ Furthermore, CNF has been used for the preparation of edible films with possible application in food packaging,^48^ membranes for water filtration and catalytic hydrogenation of dyes,^49^ aerogels for thermal insulation and fire retardancy,^50^ and coatings to provide antifouling properties to PVA filters.^51^ In this study, CNF was obtained in the form of a translucent colloidal dispersion, as shown in Figure 3. AFM (atomic force microscopy) images confirmed the nanoscale morphology with diameters in the range of 5–10 nm of the produced CNF (Figure 4).

**Figure 5:**
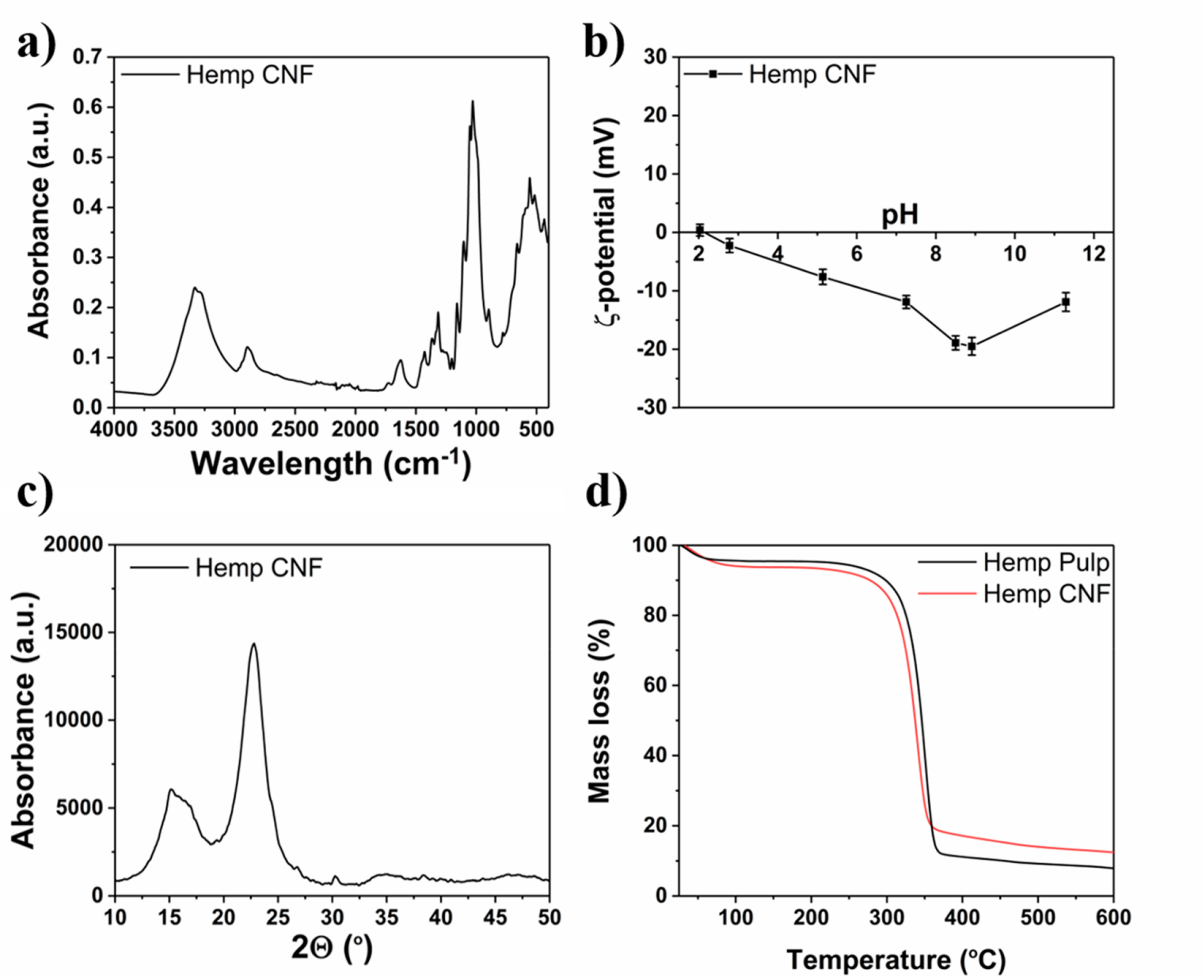
Characterization of hemp hurd CNF: (a) FTIR, (b) ζ-potential, (c) XRD, and (d) TGA of the produced CNF.

From the FTIR spectrum (Figure 5a) of the produced CNF, characteristic peaks of a stretching vibration corresponding to O–H bonds of the hydroxyl groups (3330 cm^–1^), a stretching vibration corresponding to the C–H bonds (2896 cm^–1^), and the stretching vibration of the C=O bonds of the carbonyl groups (1620 cm^–1^) can be observed.^52,53^ The presence of carbonyl groups is explained by the residual hemicellulose in the pulp after bleaching (approximately 2 wt %, as mentioned earlier), which is also confirmed by the surface charge from a conductometric titration, which showed a density of 43 mmol/kg, and the ζ-potential values, which are negative throughout a pH range between 2 and 12 (Figure 5b).^54^ The XRD diffractogram (Figure 5c) shows the characteristic peaks at 16 and 22° corresponding to the (110) and (200) Miller indices, respectively. The peak of the former index appears to be broader, which is expected in the case of CNF.^39^ The crystallinity index (*C*_rI_) of the produced CNF was calculated from the obtained diffractogram to be 79%, using the empirical method reported by Segal et al.^55^ The TGA spectra (Figure 5d) show an onset of thermal degradation at 328 °C for the hemp pulp and at 317 °C for the hemp CNF. In the case of CNF, a severe reduction in the molecular weight of the polymer chains in combination with a higher number of terminal points of the chains explain the earlier degradation onset.^56^ At the end of the degradation process, the hemp pulp showed an 87 wt % change in mass while the hemp CNF showed an 83 wt % change in mass (after the amount of water/humidity in the samples was considered). This difference and, hence, the production of more char from the CNF have been previously reported and attributed to the smaller size of the individual particles.^53^

Overall, the morphology, surface chemistry, and physical properties of the produced CNF agree with those of the conventional CNF; hence, the bleached pulp obtained in this work is an excellent candidate for the production of high-quality CNF.

## Conclusions

Valorization of hemp hurd, a byproduct from textile fiber production, was performed by RCF. The RCF was conducted in the presence of a transition-metal catalyst (Pd/C) and formic acid in MeOH/H_2_O with the addition of *p*-TSA to yield 38.8 wt % of monophenolic compounds. This corresponds to above 95% of the theoretical maximum yield. We propose that the high yield can be explained by the high S/G ratio and the thin cell wall of the hemp hurd. Organosolv pulping followed by chlorite bleaching was performed to obtain pulp in 50 wt % yield that meets the requirement for viscose production. Thus, this increases the overall yield of textile fiber production from hemp to 100%. This pulp was also successfully used to produce nanocellulose in high yields. This research discloses a new value chain of using hemp hurd, a byproduct from hemp fiber production where both the lignin and cellulose fractions have been valorized.

## Experimental Section

### Feedstock Analysis

The full feedstock analysis including extraction, two-step acid hydrolysis, HSQC, nitrobenzene oxidation, and thioacidolysis can be found in the Supporting Information

### RCF of Hemp Hurd

Raw hemp hurd (0.2 g), Pd/C 5% (20 mg), and 4 mL of MeOH/H_2_O (7/3) containing 1.1 g/L of *p*-TSA were placed in a 7 mL steel reactor, followed by addition of 80 mg of formic acid. The reaction was conducted at 200 °C. The lignin oil was extracted with DCM, washed with water, and dried over anhydrous Na_2_SO_4_. The catalyst was filtered through Celite. The collected organic phase was filtered and concentrated under reduced pressure. The crude product was dissolved in 10 mL of acetonitrile, tetracosane as an internal standard was added for GC-MS/FID analysis, and the yield of monophenolic compounds was determined to be 38.3 wt %.

### Organosolv Pulping of Hemp Hurd

The dissolving-grade pulp was prepared by placing 0.8 g of hemp hurd, 10 mL of the solvent mixture EtOH/H_2_O (65/35), and 100 μL of 1% H_2_SO_4_ in a 20 mL stainless steel reactor. The reaction mixture was heated at 175 °C for 3 h. After the reaction, the reactor was cooled to room temperature and the pulp was separated by filtration. The wood pulp was transferred into a 2 L beaker that contained 600 mL of distilled water and disintegrated with a T-25 digital ULTRA-TURRAX Homogenizer with 20.4 × 1000 rpm for 15 min. The 0.4 g of the solid pulp was obtained after filtration (50 wt % from the original hemp hurd). The obtained solid pulp was then bleached with 6.0 g pulp/600 mL of bleaching solution (300 mL of 1.7% NaClO_2_ + 300 mL of 2.7% NaOH + 7.5% AcOH). The bleaching was conducted at 80 °C for 2 h. After completion, the reaction mixture was cooled to room temperature and the bleached pulp was separated by filtration with negligible loss in weight and dried overnight for further analysis.

## Supporting Information Available

Detailed experimental procedures, analytical data, and spectra (PDF)
