# Supplementary material for: Holistic Valorization of Hemp through Reductive Catalytic Fractionation
Source: ACS Sustain Chem Eng. 2021 Dec 15;9(51):17207–13. doi: 10.1021/acssuschemeng.1c06607 (PMC8715730; doi:10.1021/acssuschemeng.1c06607)
Supplement: Supplementary file 1 — sc1c06607_si_001.pdf [file sc1c06607_si_001.pdf]

Supporting Information

Holistic Valorization of Hemp through Reductive Catalytic Fractionation

Suthawan Muangmeesri[a], Ning Li[a,b], Dimitrios Georgouvelas[c], Pierre Ouagne[d], Vincent Placet[e], Aji P. Mathew[c], Joseph S. M. Samec[a*]*

Corresponding authors: Joseph S. M. Samec, Email address- joseph.samec@su.se

Aji P. Mathew, Email address- aji.mathew@mmk.su.se

[a]Department of Organic Chemistry, Stockholm University, 106 91, Stockholm (Sweden)

[b]State Key Laboratory of Catalysis (SKLC), Dalian National Laboratory for Clean Energy (DNL), Dalian Institute of Chemical Physics (DICP), Dalian 116023, China

[c]Department of Materials and Environmental Chemistry, Stockholm University, 106 91, Stockholm (Sweden)

[d] Laboratoire Génie de Production, Université de Toulouse, ENIT, 65016 Tarbes, France

[e] Univ. Bourgogne Franche-Comté, FEMTO-ST Institute, UFC/CNRS/ENSMM/UTBM, Department of Applied Mechanics, F-25000 Besançon, France

Number of pages: 11

Number of figures: 9

Number of tables: 1

General information

The 7 mL and 25 mL stainless steel reactors used in RCF were purchased from Swagelok Company. Biomass feedstock was obtained from Eurochanvre, France. All chemicals were purchased from Fischer chemicals, CCS Healthcare AB Sweden, Sigma-Aldrich, Honeywell, and VWR chemicals, and used as received. The cellulase enzyme (Ctec 2) was purchased from Sigma-Aldrich. Enzymatic hydrolysis was performed in an incubating shaker (Forma Orbital Shaker-Model 420 Series, Thermo Electron Corporation). Sugar analysis was carried out on an Agilent 1200 Series HPLC system. GC-MS and GC-FID analyses were conducted by a QP2020 system (SHIMADZU, Japan) equipped with two parallel HP-5MS columns (30 m × 0.25 mm × 0.25 μm). GPC analysis was performed on a Prominence-i, LC-2030C system (SHIMADZU, Japan) equipped with a UV detector at 280 nm. The HSQC-NMR spectrum was recorded on a Bruker Advance (500 MHz) with a 5 mm TCI Z–Gradient (53.0 G·cm−1) cryoprobe.

Feedstock analysis

Extraction of feedstock

A two-step extraction by hot-water and 95% ethanol was performed, following the standard protocol from NREL (2008).[1] 2.13 g (oven-dry, o.d.) of raw hemp hurd was added to the extraction thimble. 200 mL of distilled water was rinsed through the Soxhlet to soaked biomass sample and collected in the receiving flask. When reflux (8 h) was completed, water was removed from the receiving flask and the biomass sample was subsequently subjected to a 95% ethanol reflux for 16 h. Then the extractive-free hemp was air dried (1.96 g, o.d.) The extractives in hemp hurd was calculated to 8.0 wt%

Two step-acid hydrolysis

A slightly modified procedure to the NREL (2008)[2] method was performed. 300 mg of extractive-free biomass sample was transferred to a pressure bottle. 3 mL of 72% sulfuric acid was added dropwise over the biomass. The pressure bottle was put into an incubating shaker at 30 ˚C for 1 h. The acid concentration was adjusted to 4 wt% by the addition of 84 mL deionized water. The bottle was placed in an autoclave at 120 ˚C for 1 h. After completion, the bottle was removed and cooled in an ice bath. The solid residue was filtered through a pre-weighed filtered

paper, where ~20 mL aqueous solution was collected into a vial. Then the residue was rinsed with 250 mL of distilled water. The solid residue was collected and dried overnight at 105 °C. After drying, the solid was weighed and transferred to a pre-weighed crucible and put in a furnace at 575 ˚C for 6 h. After completion, the crucible was cooled down to room temperature and the ash content was less than 0.2 mg.

For acid soluble lignin analysis, 1 mL of collected aqueous solution was taken and diluted with 4 mL distilled water. The diluted solution was taken and analysed by a UV-vis spectrophotometer (Varian Cary® 50 UV-Vis Spectrophotometer) at 240 nm. The lignocellulosic data is shown in Table S1.

**Summary of Lignocellulosic Data** (% Dry Matter)						
**Sample**	**Glucan**	**Xylan**	**Klason Lignin**	**Acid Soluble Lignin**	**Extractives**	**Ash**
Fine Hemp	38.4	18.0	21.1	3.7	8.0	3.2
Large Hemp	39.3	17.2	21.5	4.9	5.5	1.8

Table S1. Summary of lignocellulosic data of fine and large particles of hemp hurd.

HSQC determination of feedstock

Fine hemp hurd was homogenized in 0.7 mL of premixed DMSO-d_6/pyridine-d_5 (4:1) for NMR analysis at 25 ˚C. The acquisition time for ^{1}H and ^{13}C was 100 ms and 8 ms,

respectively, with a relaxation delay D1 of 1 s. The main cross signals from HSQC spectrum (Figure S1) include: the H units at δ_C/δ_H 129/7.2 ppm; the G units at δ_C/δ_H 110/6.9, 115/6.7, and 120/6.8 ppm for the C_2-H_2, C_5-H_5, and C_6-H_6, respectively; the S units at δ_C/δ_H 104/6.7 ppm for $C_{2/6}$-$H_{2/6}$ and 106/7.2 ppm for the $C_{2/6}$-$H_{2/6}$ with C_α carbonyl. The signals were assigned according to the publication.[3] The result shows a semi-quantitative S/G ratio of 0.79. However, this method has limitations and is not quantitative.[4] Thus, nitrobenzene oxidation was performed to confirm the S/G ratio.

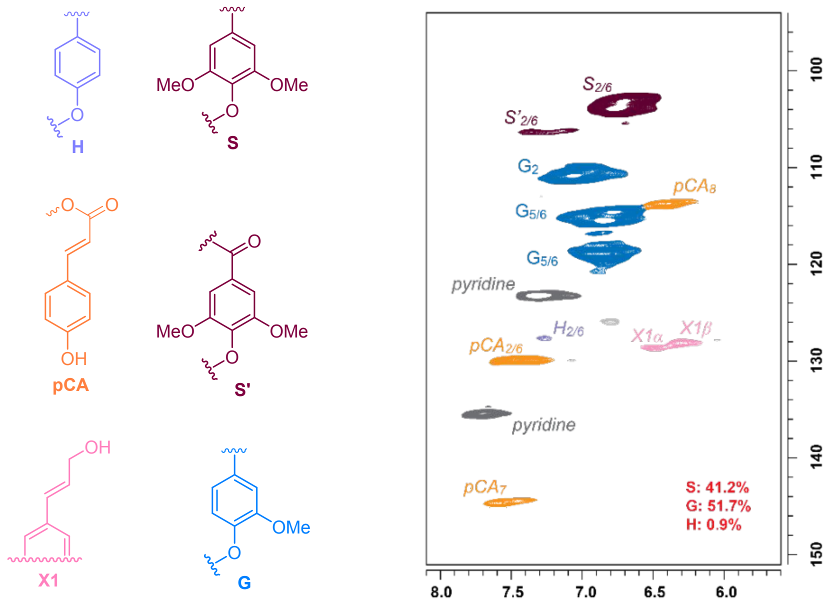

Figure S1. 2D ^{1}H-^{13}C correlation (HSQC) spectrum for hemp hurd.

Nitrobenzene oxidation

Nitrobenzene oxidation of lignin leads to the C–C bond cleavage to form vanillin and syringaldehyde (Figure S2). The S/G ratio from nitrobenzene oxidation was 0.93 quantified by GC-FID. This is higher than the 2D NMR that showed S/G ratio of 0.79.

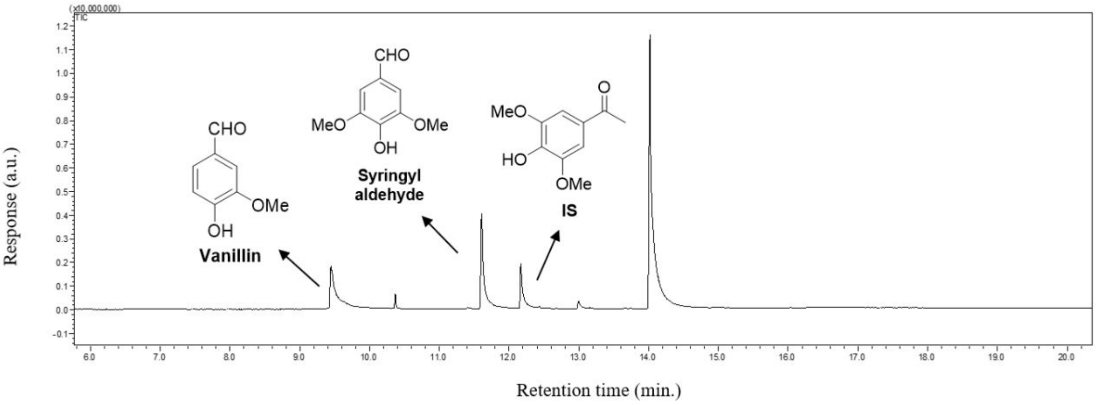

Figure S2. GC-MS spectrum of lignin-derived nitrobenzene oxidation monomers (G and S) and internal standard.

Thioacidolysis

Thioacidolysis reagent was prepared as follows; EtSH: $BF_3 \cdot Et_2O$: dioxane/ 2.5 mL: 0.7 mL: 20 mL and dioxane was added to adjust the volume to 25 mL. 40 mg of biomass was added into a seal tube followed by addition of thioacidolysis reagent (4 mL). The reaction was performed at 100 ˚C for 4 h. After completion, the reactor was cooled down in an ice-bath. Saturated $NaHCO_3$ was added to the reaction mixture followed by addition of 1M HCl until the mixture reached pH 1-3. The mixture was extracted by DCM, water, brine, and dried over

anhydrous Na_2SO_4. The organic phase was filtered and the solvent was removed under pressure. 5 mL of EtOAc was used to dissolve the resulting crude. Then 0.5 mL of the dissolved

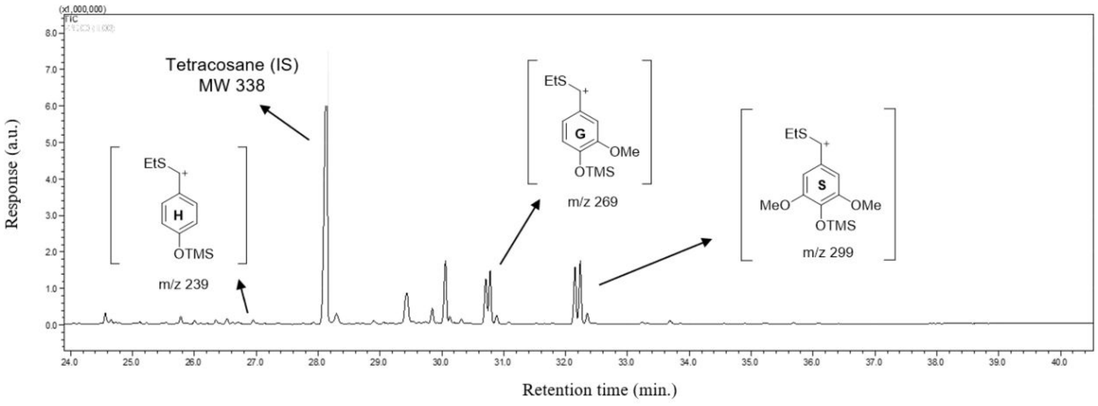

crude and 0.1 mL tetracosane in EtOAc solution (internal standard) were transferred to a 5 mL glass vial. The solvent was evaporated. 0.1 mL of BSTFA and 0.1 mL of pyridine were added to the vial and the silylation reaction was performed at 60 ˚C for 30 minutes. The solution mixture was then subjected to GC-MS/FID analysis. The results from the thioacidolysis is shown in Figure S3 with 63.0% β-O-4 bond content.

Figure S3. GC mass spectra of lignin-derived thioacidolysis monomers (G and S) and internal standard. The structures of the base peak fragment ions are shown.

Reductive catalytic fractionation

Raw hemp hurd (0.2 g), Pd/C 5% (20 mg), and 4 mL of MeOH: H_2O (7: 3) containing 1.1 g/L *p*-TSA were added in a 7 mL steel reactor. Followed by addition of 80 mg of formic acid. The reaction was conducted at 200˚C. The lignin oil was extracted by DCM, washed by water, and

dried over anhydrous Na_2SO_4. The catalyst was filtered through celite. The collected organic phase was filtered and concentrated under reduced pressure. The crude was dissolved in 10 mL of acetonitrile and tetracosane as internal standard was added for GC-MS/FID analysis. The results are shown in Figure S4, and show peaks that correspond to 38.3 wt% yield (means value of three repetitions; 40.0, 38.2, 36.7 wt%) of monophenolic compounds under the optimized condition.

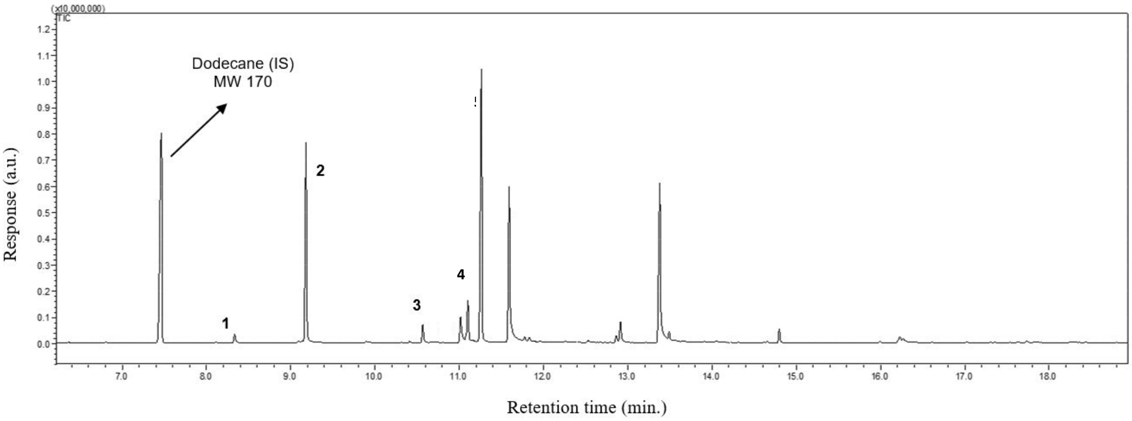

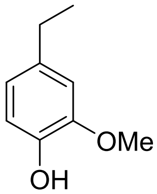

1

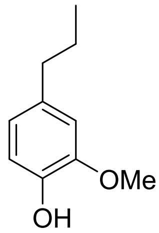

2

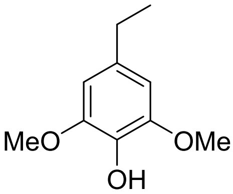

3

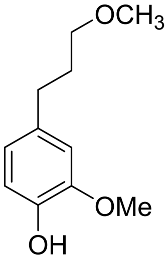

4

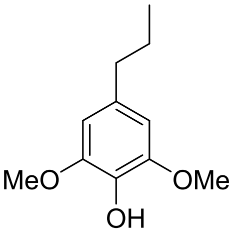

5

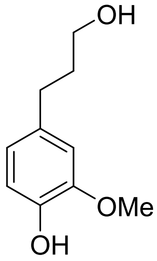

6

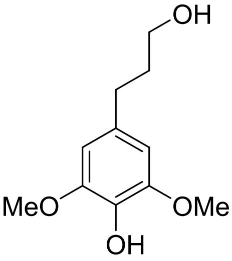

7

Figure S4. GC mass spectra and monolignols from RCF

GPC

GPC (Gel permeation chromatography) provides a semi-quantitative estimation of the molecular weight distribution of the lignin oil (Figure S5). The 1 hour run has higher amounts of oligomers (especially large fragments >529 Da, at 23.25 min) than dimers and monomers due to the incomplete lignin depolymerization. Increasing the reaction time led to a

significantly lower amount of oligomers (at 4 hours run). It should be noted that the peak data after 25 min of retention time were fluctuated due to machine sensitivity and not considered for the analysis.

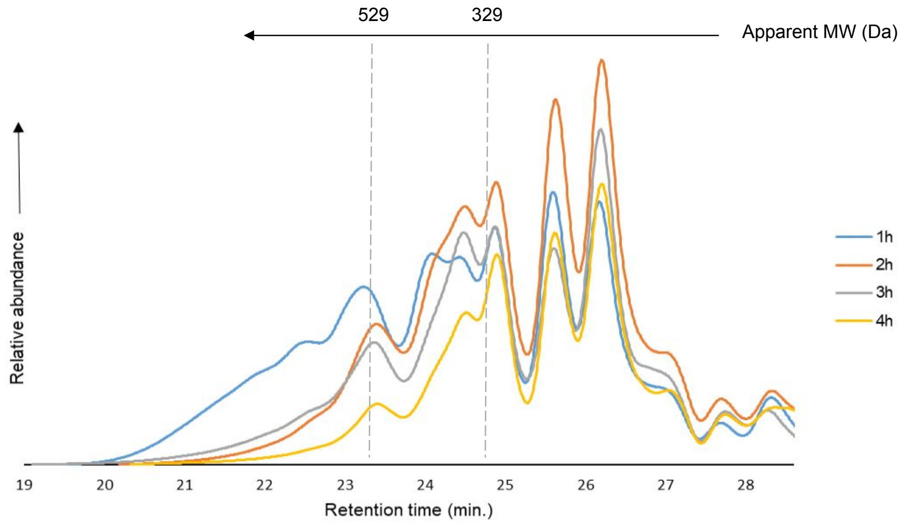

Figure S5. GPC chromatogram for molecular weight distribution of products after RCF

Enzymatic hydrolysis

0.2 g of pulped hemp hurd and 5 mL of buffer solution (NaOAc, 10mM, pH= 4.8) were added into an Erlenmeyer flask. The total volume was adjusted to 10 mL by addition of DI water. The mixture was stirred in an incubating shaker at 50˚C for 30 minutes. Cellulase enzyme, CTec2 (20 FPU/g substrate) was added afterward. The mixture was placed in an incubating shaker at 50˚C for 48 h. At the end of the hydrolysis, the solid residue was filtered, air-dried, and the aqueous solution was diluted 20× for HPLC analysis.

Dissolving grade pulp

The dissolving grade pulp was prepared by 0.8 g of biomass (hemp hurd), 10 mL of solvent mixture EtOH: H_2O (65:35), 100 μL of 1% H_2SO_4 in 20 mL stainless steel reactor, and the reaction was conducted at 175˚C for 3h. After the reaction, the reactor was cool to room temperature. The pulp was separated by filtration. The wood pulp was transferred into a 2L beaker that contains 600 mL distilled water and disintegration of the pulp followed by homogenizing the pulp sample with T-25 digital ULTRA-TURRAX® Homogenizer with 20.4 × 1000 rpm for 15 min. The solid pulp was obtained after filtration (50 wt% from the original pulp). The obtained solid pulp was then bleached with a bleaching solution. The reaction was conducted at 80˚C for 1-2h. After completion, the reaction was cooled to room temperature and the bleached pulp was separated by filtration and dried overnight for further analysis.

Bleaching condition: 6.0g pulp/600mL of bleaching solution (300 mL of 1.7% $NaClO_2$ + 300 mL of 2.7% NaOH+7.5% AcOH).

Organosolv lignin characterization

The lignin fraction after organosolv was collected extracted with DCM, water, and dried with Na_2SO_4 anhydrous. Concentrated in vacuum and used for further analysis.

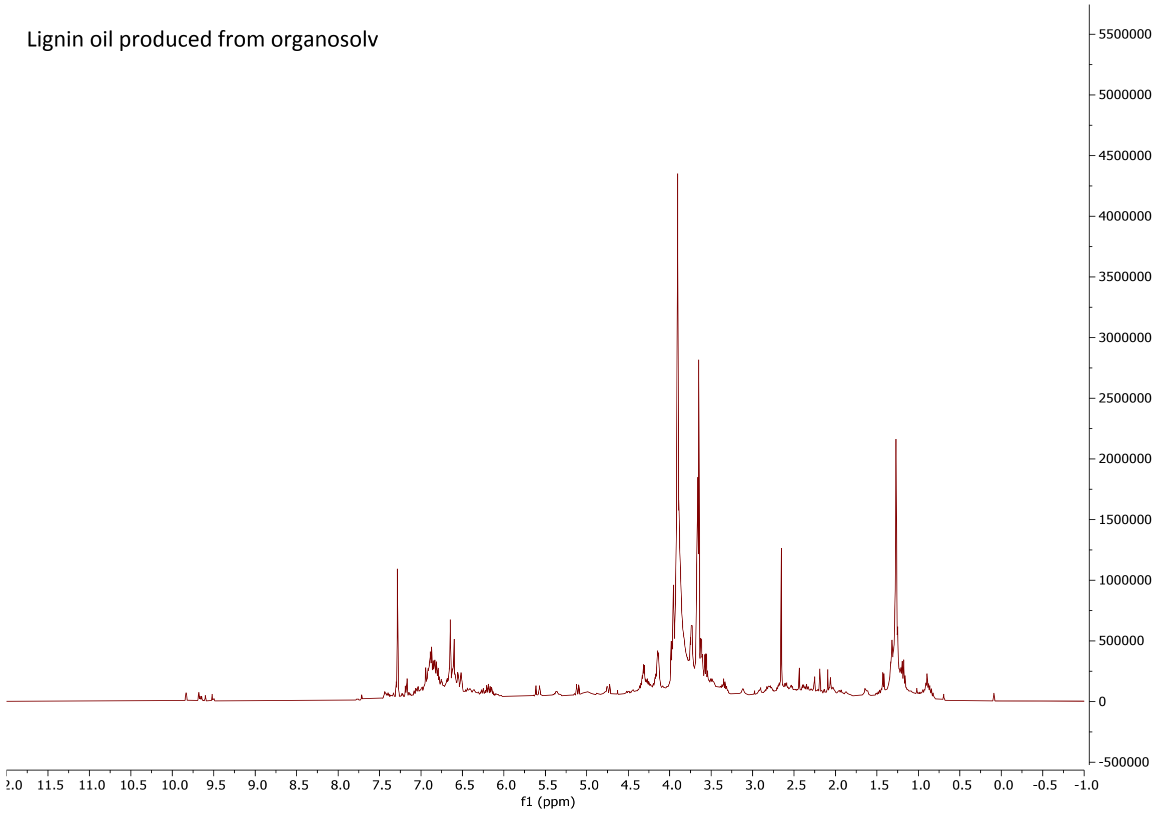

Figure S6. ^{1}H NMR spectrum of lignin oil from organosolv.

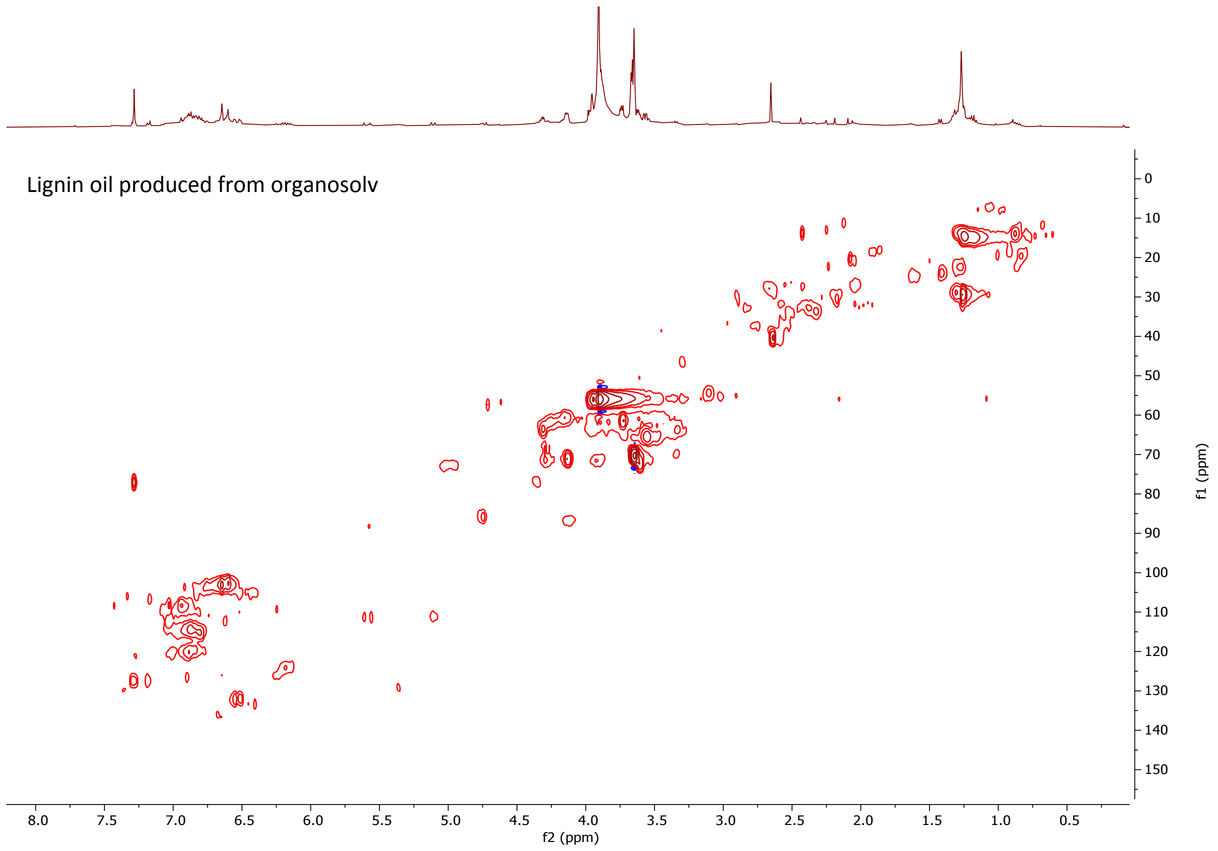

Figure S7. ^{1}H ^{13}C HSQC NMR spectrum of lignin oil from organosolv.

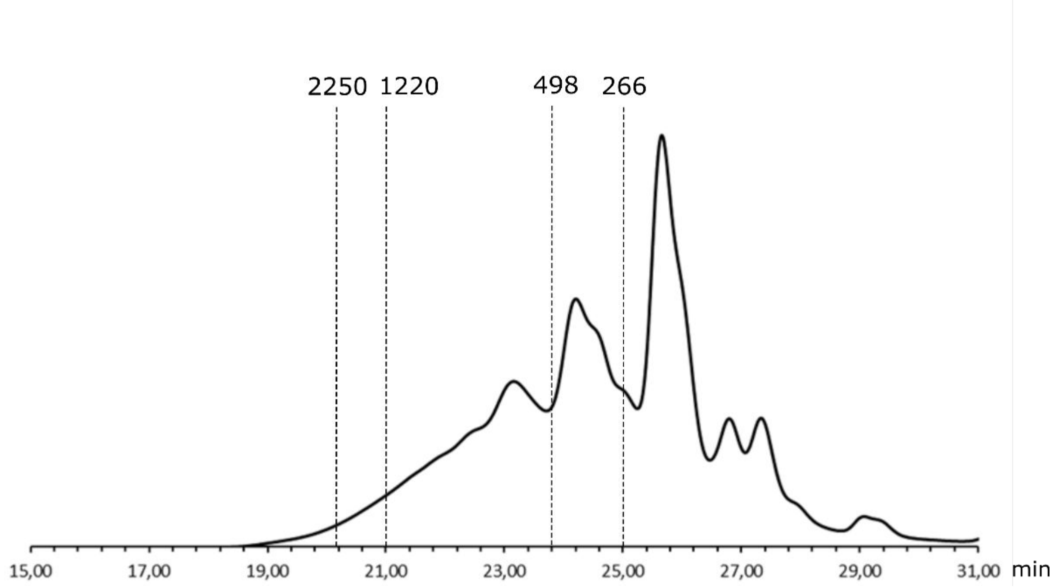

Figure S8. GPC chromatogram of lignin oil from organosolv.

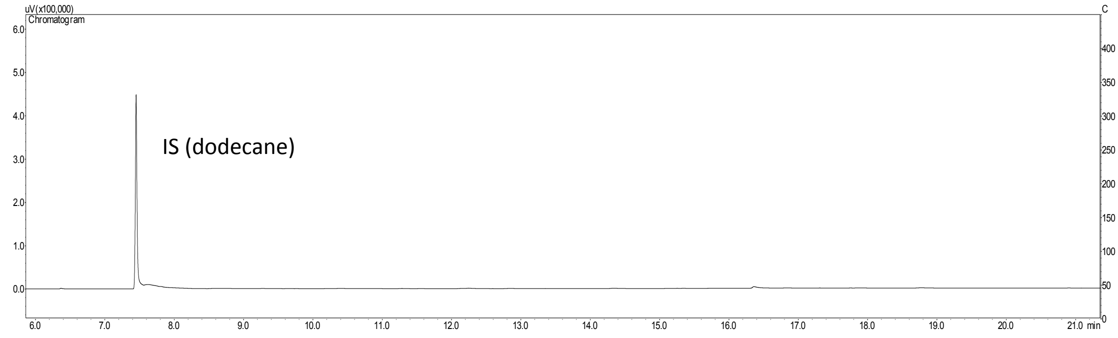

Figure S9. GC-MS chromatogram of lignin oil from organosolv with internal standard.

ISO-brightness test

The ISO–brightness test was performed on a bleached hemp hurd pulp sheet by using standard method *ISO 2470-1* which gave the brightness value of 90.2%.

Degree of polymerization (DP) measurements

The degree of polymerization (DP) value is 898 according to method *SCAN-C 15:62.*

Nanocellulose

The bleached hemp pulp was first subjected to vigorous stirring with UltraTurrax at 15000 rpm for 1 h in order to break down big agglomerates. Following, a 0.1 wt% dispersion of the pulp in water was prepared and passed through a homogenizer (SPXflow, APV 2000 ATEX) 10 times at 500 bar pressure.

The produced CNF was characterized with Fourier-Transform infrared spectrometry (FTIR) (PerkinElmer Spectrum 2000), and conductometric titration with NaOH to study the

functional groups and surface charge density of the material, ζ-potential (Zetasizer Nano ZS) to study the surface charge and the colloidal stability of the dispersion, X-ray diffraction (XRD) (PANalytical X'Pert PRO) to study the crystallinity, thermogravimetric analysis (TGA) (TA Instruments Discovery) in nitrogen atmosphere to study the thermal degradation, and atomic force microscopy (AFM) (Veeco Multimode V) for imaging of the fibers.

References

(1) Sluiter, A.; Ruiz, R.; Scarlata, C.; Sluiter, J.; Templeton, D. Determination of Extractives in Biomass; Laboratory Analytical Procedure (LAP). *Natl. Renew. Energy Lab.* **2008**; pp 1–9.

(2) Sluiter, A.; Hames, B.; Ruiz, R.; Scarlata, C.; Sluiter, J.; Templeton, D.; Crocker, D. Determination of Structural Carbohydrates and Lignin in Biomass, in: Laboratory Analytical Procedure (LAP). *Natl. Renew. Energy Lab.* **2008**; pp 1-18.

(3) Li, N.; Pan, X.; Alexander, J. A Facile and Fast Method for Quantitating Lignin in Lignocellulosic Biomass Using Acidic Lithium Bromide Trihydrate (ALBTH). *Green Chem.* **2016**, *18* (19), 5367–5376. https://doi.org/10.1039/C6GC01090C.

(4) Ralph, J.; Marita, J. M.; Ralph, S. A.; Hatfield, R. D.; Lu, F.; Ede, R. M.; Peng, J.; Landucci, L. L. Solution State NMR of Lignins. *Adv. Lignocellul. Charact. Atlanta Ga TAPPI Press. 55-108 Ill* **1999**.
